# Significant but Temporary Efficacy of Statin for a Patient With Severe Autoimmune Pulmonary Alveolar Proteinosis: A Case Report

**DOI:** 10.1002/rcr2.70507

**Published:** 2026-02-17

**Authors:** Fumihiko Makino, Kohei Shibayama, Yuki Muto, Rie Hayakawa, Kenta Izumi, Yohei Suzuki, Osamu Nagashima, Shinichi Sasaki, Kazuhisa Takahashi

**Affiliations:** ^1^ Department of Respiratory Medicine Juntendo University Urayasu Hospital Chiba Japan; ^2^ Department of Respiratory Medicine Faculty of Medicine and Graduate School of Medicine, Juntendo University Tokyo Japan

**Keywords:** anti‐GM‐CSF antibody, autoimmune pulmonary alveolar proteinosis, dyslipidaemia, foamy alveolar macrophages, oral statin therapy

## Abstract

Autoimmune pulmonary alveolar proteinosis (APAP) is a rare autoimmune lung disorder characterised by the presence of anti‐granulocyte‐macrophage colony‐stimulating factor (GM‐CSF) antibodies. Whole‐lung lavage (WLL) therapy remains the standard treatment for severe cases. Recently, inhaled GM‐CSF therapy has been approved in Japan; however, the cost of the treatment remains a limiting factor. Several reports have suggested that oral statin therapy may be a promising therapeutic option for APAP. Herein, we report a case of severe APAP that underwent WLL therapy twice and achieved an excellent response and remarkable clinical resolution of respiratory failure after the initiation of oral statin therapy. Remarkable improvements in oxygen saturation, blood gas analysis, serum biomarker levels and pulmonary function test results were observed after statin administration. However, the efficacy was temporary, and respiratory failure relapsed 2 years after the initiation of statin therapy. Statin therapy for APAP was deemed effective but potentially temporary.

## Graphical abstract

Evidence of the efficacy of statins in pulmonary alveolar proteinosis is limited and remains controversial. We report a case of severe autoimmune pulmonary alveolar proteinosis in a patient who underwent whole‐lung lavage therapy twice and achieved excellent response and remarkable clinical resolution of respiratory failure after the initiation of oral statin therapy.

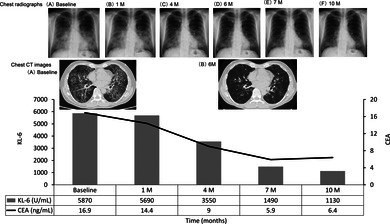

## 1 Introduction

Pulmonary alveolar proteinosis (PAP) is a rare pulmonary disorder characterised by progressive respiratory failure due to excessive accumulation of surfactant proteins and lipids in the alveoli and peripheral airways. PAP is classified into three types based on the aetiology: autoimmune PAP (APAP), secondary PAP and congenital/hereditary PAP. Among these, APAP is the most common, accounting for approximately 92% of all cases. Additionally, APAP is caused by the presence of anti‐granulocyte‐macrophage colony‐stimulating factor (GM‐CSF) antibodies, which disrupt the GM‐CSF signalling pathway. This impairment disturbs the maturation and functional maintenance of alveolar macrophages [1].

The pathophysiology of APAP involves the impaired clearance of surfactant in the alveoli as a result of reduced GM‐CSF‐dependent cholesterol mechanism in alveolar macrophages [1, 2, 3].

Whole‐lung lavage (WLL) therapy is the standard treatment for severe cases of PAP and is highly effective [1]. However, the treatment modality is invasive and can only be performed in limited facilities. Recently, inhaled GM‐CSF was approved in Japan as a viable option for APAP. However, the high cost of APAP remains a significant concern.

Evidence on statin efficacy in PAP is limited and remains controversial [4, 5]. In this report, we present a case of APAP that recovered from respiratory failure following statin therapy and explore the potential role of statins in PAP management.

## 2 Case Report

The patient, a 60‐year‐old man, was diagnosed with APAP at a prior hospital 2 years ago. At the initial visit, he presented with progressive dyspnoea on exertion (DOE). Chest radiography during a routine health checkup revealed ground‐glass opacities (GGO) in both lungs. Computed tomography demonstrated a crazy‐paving pattern, while bronchoscopy revealed white, milk‐like bronchoalveolar fluid (BALF). Blood tests revealed anti‐GM‐CSF antibodies at a level of 46.9 μg/mL, confirming the diagnosis of APAP. His condition gradually worsened. He underwent four partial lung lavages 16 months ago, followed by one unilateral lung lavage 6 months ago. The patient was transferred to our hospital due to relocation.

At the initial visit to our hospital, he presented with chronic respiratory failure, an oxygen saturation of 89% at room temperature and dyspnoea. Home oxygen therapy was not initiated, as the patient declined treatment. Blood tests revealed abnormalities in lipid metabolism (high low‐density lipoprotein [LDL] cholesterol, 204 mg/dL); therefore, statin pitavastatin calcium therapy (1 mg/day) was initiated.

**FIGURE 1:**
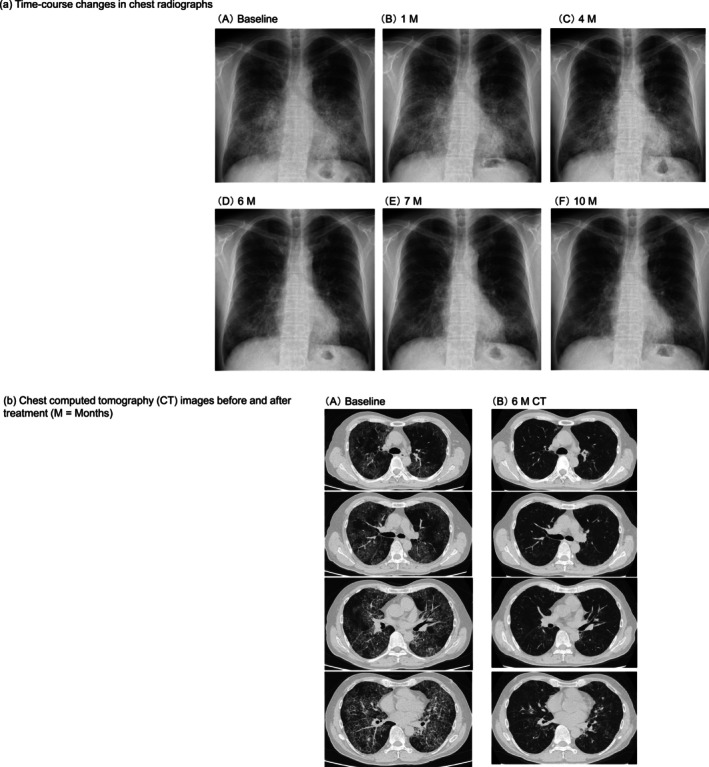
Sequential radiological imaging findings (M = Months). (a) Time‐course changes in chest radiographs at the following time‐points: (A) baseline, (B) 1 M, (C) 4 M, (D) 6 M, (E) 7 M and (F) 10 M. (b) Chest computed tomography (CT) images before and after treatment (M = Months). (A) Baseline and (B) 6 M CT.

**FIGURE 2:**
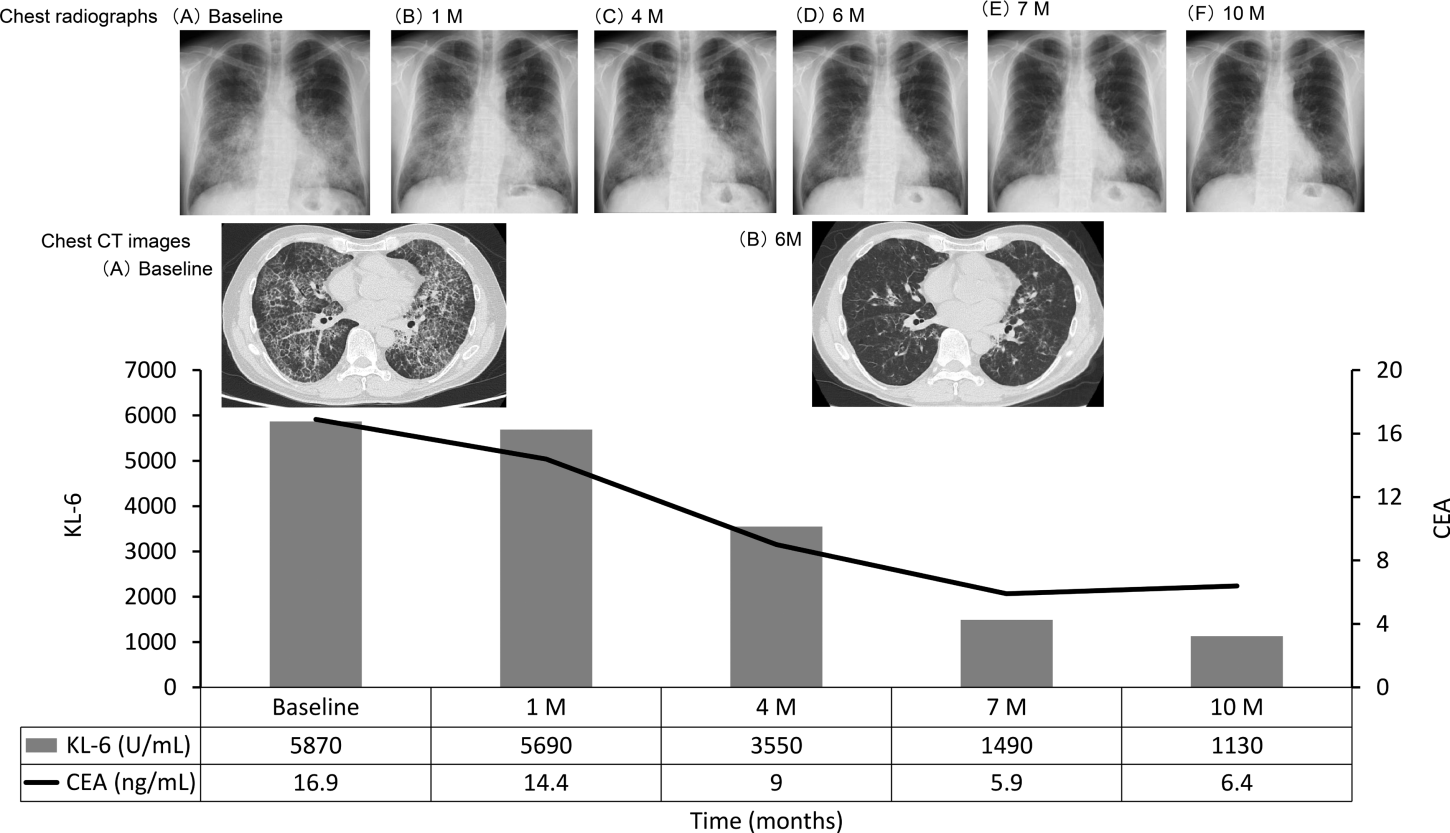
Time‐course changes in biomarkers and radiological images (M = Months). Chest radiographs at the following time‐points: (A) baseline, (B) 1 M, (C) 4 M, (D) 6 M, (E) 7 M and (F) 10 M.

One month after initiating statin therapy, not only did LDL cholesterol levels improve, but lung injury biomarkers (Krebs von den Lungen‐6, surfactant protein D, lactate dehydrogenase and carcinoembryonic antigen) also decreased. Additionally, a trend toward improvement in DOE was observed. In response, we increased the statin dose from 1 to 2 mg to further reduce lung injury caused by protein deposition in macrophages. Following statin dose escalation, the GGO areas on chest radiographs and CT scans demonstrated further improvement (Figures 1 and 2). One year after initiating statin therapy, the GGO on CT had nearly completely resolved. Oxygen saturation increased from 89% to 97% at room temperature, with the blood gas analysis confirming recovery from chronic respiratory failure (Table 1b). The elevated biomarkers returned to normal within 10 months (Table 1a), and pulmonary function tests demonstrated improvements in mixed ventilation dysfunction and diffusion impairment (Table 2). Ultimately, statin therapy resulted in the remission of PAP in the patient.

**TABLE 1:** Time‐course changes in blood test values.

(a) Disease activity markers					
Biomarker	Baseline	1‐month follow‐up	4‐month follow‐up	7‐month follow‐up	10‐month follow‐up
KL‐6 (U/mL)	5870	5690	3550	1490	1130
SP‐D (ng/mL)	330	244	238	125	111
LDH (IU/L)	260	231	183	178	166
CEA (ng/mL)	16.9	14.4	9.0	5.9	6.4

(b) Blood gas analysis (BGA)		
BGA (Room air)	Baseline	8‐month follow‐up
pH	7.421	7.404
PaCO_2_ (mmHg)	33.5	36.6
PaO_2_ (mmHg)	60.0	91.7
HCO_3_^−^ (mmol/l)	21.4	22.4
BE (mmol/l)	−1.6	−1.9

Abbreviations: CEA, carcinoembryonic antigen; KL‐6, Krebs von den Lungen; LDH, Lactate dehydrogenase; SP‐D, Surfactant protein D.

**TABLE 2:** Changes in pulmonary function test volumes and percentage predicted values.

Pulmonary function test	Baseline	7‐month follow‐up
VC (L)	3.05	3.63
FEV1 (L)	2.35	2.65
FVC % pred (%)	72.1	87.0
FEV1% pred (%)	68.3	78.3
DLCO % pred (%)	54.6	61.4

Abbreviations: DLCO, carbon monoxide diffusion capacity; FEV1, forced expiratory volume in 1 s; FVC, forced vital capacity; VC, vital capacity.

However, the therapeutic efficacy of statin therapy appears to be transient. Approximately 2 years after beginning statin therapy, APAP aggravated again, leading to respiratory failure. Lung injury biomarkers increased again, with the following values: KL‐64600 U/mL, SP‐D 444 ng/mL, LDH 252 IU/L and CEA 25 ng/mL. Hence, the patient was transferred to another hospital for re‐administration of WLL.

## 3 Discussion

In this case, the patient demonstrated significant improvement in type I respiratory failure within 6–12 months after initiating statin therapy; however, the efficacy of therapy was not sustained. Unfortunately, the worsening of autoimmune PAP could not be avoided 2 years after the initiation of statin therapy. For hyperlipidaemia, statin therapy was initiated along with lifestyle counselling for dietary fat restriction. This resulted in notable improvement of the APAP. Paradoxically, this improvement may have caused the patient to let their guard down. Although medication adherence remained intact, the patient completely discontinued fat restriction and overindulged in food and drink as a rebound effect. This may have ultimately led to marked worsening of the APAP.

ATP‐binding cassette transporters ABCA1 and ABCG1 mediate cholesterol efflux from alveolar macrophages [1, 2, 3, 5]. In PAP, the messenger ribonucleic acid (mRNA) transcription levels of ABCA1 and ABCG1 are reduced [1, 2, 3, 5]. This results in impaired GM‐CSF‐dependent cholesterol clearance by alveolar macrophages, leading to foamy body formation accompanied by excessive free and esterified cholesterol. Cormac et al. reported that statins increase the mRNA levels of ABCA1 and ABCG1 by inhibiting 3‐hydroxy‐3‐methylglutaryl‐CoA reductase, thereby increasing the expression of sterol regulatory element‐binding protein‐2 in alveolar macrophages and promoting the clearance of pulmonary surfactants [1, 5].

Previous reports on autoimmune PAP have confirmed that the efficacy of statins lasts for approximately 1 year. Reports from China have also demonstrated that statins are effective in patients with APAP without dyslipidaemia; however, their efficacy is short‐lived. This may be because the presence of anti‐GM‐CSF antibodies induces the continuous formation of foamy macrophages through an autoimmune reaction, which may outweigh the benefits of statin therapy.

As the production of foamy macrophages induced by autoantibodies cannot be inhibited, disease progression is presumed to occur once intracellular lipid accumulation exceeds the improvement in cholesterol metabolism in alveolar macrophages achieved by statin therapy. Therefore, GM‐CSF supplementation is considered the only treatment with the potential to provide long‐term therapeutic benefits.

Traditionally, the standard treatment for severe PAP has been WLL; however, inhaled GM‐CSF has recently been approved in Japan as a new option for APAP. Compared to conventional WLL, inhaled GM‐CSF therapy is less invasive and can be administered at home; however, its high cost is a concern. Statins could serve as an effective treatment option to bridge the gap between WLL and inhaled GM‐CSF for severe APAP.

## Author Contributions

Fumihiko Makino was responsible for patient management and drafting the initial manuscript. Kohei Shibayama, Yuki Muto, Rie Hayakawa, Kenta Izumi, Yohei Suzuki and Osamu Nagashima reviewed the clinical course and laboratory findings, interpreted the radiological images and critically revised the manuscript. Shinichi Sasaki and Kazuhisa Takahashi supervised the case study and approved the final version of the manuscript. All the authors contributed equally to the discussion of the results and approved the final version of the manuscript.

## Funding

The authors have nothing to report.

## Consent

The authors declare that written informed consent was obtained for the publication of this manuscript and accompanying images using the consent form provided by the journal.

## Conflicts of Interest

K.T. is an Editorial Board member of Respirology Case Reports and a co‐author of this article. He was excluded from all editorial decision‐making related to the acceptance of this article for publication. The other authors declare no conflicts of interest.

## Data Availability

Data sharing not applicable to this article as no datasets were generated or analysed during the current study.
